# Maintenance of postharvest storability and overall quality of ‘Jinshayou’ pummelo fruit by salicylic acid treatment

**DOI:** 10.3389/fpls.2022.1086375

**Published:** 2023-01-11

**Authors:** Qiang Huang, Lulu Huang, Jinyin Chen, Yajie Zhang, Wenbin Kai, Chuying Chen

**Affiliations:** Jiangxi Key Laboratory for Postharvest Preservation and Non-Destruction Testing of Fruits & Vegetables, College of Agriculture, Jiangxi Agricultural University, Nanchang, China

**Keywords:** *Citrus maxima* Merr., salicylic acid, postharvest storablitiy, overall quality, ROS homeostasis

## Abstract

**Introduction:**

The loss of postharvest storability of pummelo fruit reduces its commodity value for long run. To maintain its storability, the effects of postharvest dipping treatment by salicylic acid (SA) with different concentrations (0, 0.1, 0.2, or 0.3%) were investigated on pummelo fruit (*Citrus maxima* Merr. cv. Jinshayou) during the room temperature storage at 20 ± 2°C for 90 d.

**Results and discussion:**

Among all treatments, pre-storage SA treatment at 0.3% demonstrated the most significant ability to reduce fruit decay incidence, decrease weight loss, delay peel color-turned process, and inhibit the declines in total soluble solids (TSS) as well as titratable acid (TA) content. The increases in electrolyte leakage, hydrogen peroxide (H_2_O_2_), and malondialdehyde (MDA) content of the 0.3% SA-treated pummelo fruit were reduced compared to the control (dipped in distilled water). Pummelo fruit treated with 0.3% SA exhibited the most outstanding ability to excess reactive oxygen species (ROS) accumulation, as evidenced by promoted the increases in glutathione (GSH), total phenolics and flavonoids contents, delayed the AsA decline, and enhanced the activities of antioxidant enzymes and their encoding genes expression.

**Conclusion:**

Pre-storage treatment dipped with SA, particularly at 0.3%, can be used as a useful and safe preservation method to maintain higher postharvest storability and better overall quality of ‘Jinshayou’ pummelo fruit, and thus delaying postharvest senescence and extend the storage life up to 90 d at room temperature.

## 1 Introduction

Pummelo (*Citrus maxima* Merr.) fruit, the largest known citrus fruit, is a non-climacteric subtropical fruit belonging to the *Citrus* family, and widely cultivated in Southeast China (e.g., Fujian, Jiangxi, Zhejiang, Guangdong, and other adjoining provinces) (Chen et al., 2021; Chen et al., 2022). Due to its rich nutrients (e.g., dietary fiber, organic acids, vitamins, pectins, flavonoids and minerals), full succulency, attractive appearance, pleasant flavor, and blessed moral, pummelo fruit is considered as a highly appreciated citrus fruit by consumers. Pummelo fruit has a huge shape and large weight, which is an enormous challenge during the postharvest periods, particularly during transportation and storage, leading into a remarkable restriction to the marketability and industrial development. Several preservation methods have been applied to maintain postharvest storability of harvested pummelo fruit, such as CaCl_2_ (Chen et al., 2005), chitosan (Nie et al., 2020; Chen et al., 2021), gibberellic acid (Porat et al., 2001), and 1-methylcyclopropene (Lacerna et al., 2018). As the closest citrus fruit to pummelo fruit, grapefruit can be effectively preserved by using several postharvest approaches, including hot water, biocontrol agent GS-3, neem leaf extract, and pectic oligosaccharides (Porat et al., 2000; Vera-Guzmán et al., 2019; Deng et al., 2020; Khan et al., 2021). Due to the long-established application of postharvest methods for pummelo, and the dissimilarities between the above two citrus varieties, the development of effective postharvest technologies for pummelo preservation is of great importance and urgency.

Salicylic acid (SA), an important plant phenolic compound, acts as an important signaling molecule with a role in enhancing postharvest resistance to senescence stress and pathogen invasion in horticultural products during long-term storage and shipment (Belay and James Caleb, 2022). Recently, SA treatment applied for postharvest purposes to delay fruit senescence has attracted increasing attention. Numerous studies have demonstrated that pre-storage treatment with SA effectively alleviates firmness loss and quality deterioration in mandarin fruit (Haider et al., 2020), lemon fruit (Serna-Escolano et al., 2021), apricot fruit (Li et al., 2022), bell pepper (Ge et al., 2020), grapefruit (Shi et al., 2019), longan fruit (Chen et al., 2020), winter jujube (Sang et al., 2022), and pear fruit (Sinha et al., 2022). In these studies, the maintenance of postharvest storability in horticultural fruits was deemed to be related to phytochemicals and enzymes involved in cell wall metabolism, reactive active oxygen (ROS) metabolism, as well as energy metabolism. For example, Baswal et al. (2020) found that 2 μM SA could extend the cold-stored life of ‘Kinnow’ mandarin up to 75 d by reducing firmness loss, alleviating nutritional quality (ascorbic acid, carotenoids, soluble sugars and titratable acids) deterioration and delaying cell wall-softening enzymes activities. In addition, 2 mM SA treatment reduced postharvest decay and pulp browning of ‘Patharnakh’ pear by maintaining fruit firmness and nutritional quality, inhibiting fruit respiration, and enhancing the antioxidant system, whereas the high browning index correlated to the high PPO activity and the low total phenolics content (TPC) was observed in the control fruit (Adhikary et al., 2021). Furthermore, postharvest SA application is proved to be beneficial for enhancing nutritional quality and antioxidant potential of berry fruits (cornelian cherry, blueberry, and tomato) in the period of storage (Dokhanieh et al., 2013; Kumar et al., 2021; Jiang et al., 2022).

Although pre-storage SA treatment has been shown to be useful for the postharvest preservation of various fruits and vegetables, as far as we know, there is rarely information on the alleviatory effects of SA on pummelo postharvest senescence and the overall quality of ‘Jinshayou’ pummelo fruit during long-term storage. The aim of this study was to evaluate the preservative effect of SA on postharvest fruit quality, oxidative stress, antioxidant capacity, and ROS-scavenging system in ‘Jinshayou’ pummelo fruit during the room temperature storage, and provide theoretical evidence to develop SA as a green and efficacious preservative for postharvest pummelo fruit.

## 2 Material and methods

### 2.1 Chemicals

SA, sodium hydroxide (NaOH), thiobarbituric acid (TBA), hydrochloric acid (HCl), trichloroacetic acid (TCA), methanol, ethanol, aluminum chloride (AlCl_3_), polyvinylpyrrolidone (PVP), and iron sulfate were purchased from Sinopharm Chemical Reagent Co. Ltd. (Shanghai, China). Folin–Ciocalteu reagent, gallic acid (GA), rutin, *L*-ascorbic acid (*L*-AsA), 2,2-diphenyl-1-picrylhydrazyl (DPPH), and dithiothreitol (DTT) were obtained from Sigma-Aldrich, Chemical Co. (Louis, MO, USA).

### 2.2 Fruit materials and SA treatment

Pummelo (*C. maxima* Merr. cv. ‘Jinshayou’) fruit, at mature-green stage (about 195 d after anthesis), were harvested from a commercial orchard in Ji’shui city (Jiangxi Province, China). Fruit with uniformity of weight, shape, color, maturity (firmness: 54.5–56.2 N; ripening index: 20.6–21.3), and without visible defects were selected, washed with sterile water, and randomly divided into four groups (n = 4, 240 fruit in total per treatment). Each group was dipped in 0 (control), 0.1%, 0.2%, 0.3% SA solution for 5 min. After being naturally air-dried, each fruit was individually film packaged, and stored at room temperature condition [20 ± 2°C, relative humidity (RH): 70 – 85%] for 90 d. Fruit color change, decay rate, weight loss, and other biochemical quality parameters were measured at 15 d intervals. For each sample, the tissues of juice sacs (n = 3, 10 fruit in total per replicate) randomly taken from the control and three SA-treated groups were frozen in liquid nitrogen, ground into powder and then stored at -80°C.

### 2.3 Measurement of peel hue angle (h°) and color difference (ΔE)

The CIE parameters of L* (dark to light), a* (green to red) and b* (blue to yellow) on two opposite equatorial sites of ‘Jinshayou’ pummelo peel were measured directly using a CR-400 colorimeter (Minolta Co., Osaka, Japan) following the protocol described by Mitalo et al. (2022). Both hue angle (h°) and color difference (ΔE) were calculated by the following two Hunter lab equations:

h° = arctan (b*/a*)


$$\text{ΔE = }\sqrt{{{\text{(L}}^{*}\;-\;{\text{L}}_{0}^{*})}^{2}\;+\;{{\text{(a}}^{*}\;-\;{\text{a}}_{0}^{*})}^{2}\;+\;{{\text{(b}}^{*}\;-\;{\text{b}}_{0}^{*})}^{2}}$$

Where L_0_, a_0_, and b_0_ were the readings at harvest (0 d), and L*, a*, and b* were the readings of each sampling time during storage period.

### 2.4 Evaluation of fruit decay incidence

The detailed method for evaluation of fruit decay rate has been previously described by Huang et al. (2021). The rate of fruit decay was evaluated as the number of decayed pummelo fruit with visible symptoms of pitted peel or pathogen incidence compared to the total number of pummelo fruit, and it was expressed as a percentage (%) after each storage period.

### 2.5 Determination of fruit weight loss

At 15-day intervals during storage at 20 ± 2°C, the weight loss of ‘Jinshayou’ pummelo fruit was recorded according to the protocol described by Baswal et al. (2020). The percentage (%) of WL rate was calculated compared to the initial fruit weight.

### 2.6 Assays of biochemical quality parameters

Total soluble solid (TSS) content in pummelo juice extracted from 10 fruit was determined with a digital refractometer (model: RA-250WE, Atago, Japan), calibrating with deionized water before each reading, and the results were expressed as a percentage (%). Titratable acidity (TA) content was analyzed in terms of citric acid by adding 4.0 grams extracted juice with two drops of 1% phenolphthalein in 40 mL of distilled water, and then it was titrated with 0.1 mol L^-1^ NaOH solution, and the results were calculated based on the NaOH consumption and expressed as %.

To estimate cell membrane permeability, electrolyte leakage (EL) was determined using a DDS-307A conductivity meter (Shanghai Rex., China) as described by Huang et al. (2021). The flesh tissue (5.0 grams) of juice sacs was stripped off, and then submerged in 40 mL of deionized water. Upon shaking the solution at 25°C for 30 min, the initial value (C0) was measured; subsequently, the final one (C1) was taken after the solution was exposed to a boiling water bath for 10 min. EL was reported as % from the ratio of C0 to C1.

For hydrogen peroxide (H_2_O_2_) content assay, a total of 2.0 g frozen sample was extracted with 5 mL of pre-cooled acetone and the homogenate was centrifuged (10 000 × *g* at 4°C for 20 min) to discard the residue. H_2_O_2_ content was determined using a specific detection kit (No: BC3590, Solarbio, Beijing, China) by monitoring the absorbance at 412 nm (Ackah et al., 2022), with the results reported as micromole per gram (μmol g^-1^) on a frozen weight (FW).

Malondialdehyde (MDA) content was assayed using the TBA method as described by Bakpa et al. (2022) with a slight modification. Briefly, 2.0 g of frozen juice sac was extracted with 5 mL of 10% (*m/v*) TCA solution and centrifuged (10 000 × *g* at 4°C for 20 min). Afterwards, 2.0 mL of the supernatant was mixed by adding the same volume of 0.67% TBA (dissolved in 50 mM NaOH) solution, followed by boiling water bath for 20 min, and then quickly cooled in an ice bath. Finally, the absorbance of the supernatant was recorded at three specific wavelengths (450 nm, 532 nm, and 600 nm) using a UV-Vis spectrophotometer (model: TU-1950, Persee General Instrument Co., Ltd., Beijing, China), with the results were reported as millimole per gram (mmol g^-1^) FW.

Quantitative determination of AsA content in pummelo fruit was carried out on juice sac samples according to the 2,6-dichlorophenol–indophenol (DPIP) dye titration method described by Huang et al. (2021), with *L*-AsA as the standard, where the AsA content was expressed as mg of AsA equivalent per 100 g of juice sac FW.

The glutathione (GSH) content of pummelo juice sac was determined using the 5,5’- dithiobis-(2-nitrobenzoic acid) reaction method, a protocol described by Nie et al. (2020), with 412 nm as the target wavelength, where the GSH content was reported as milligram per kilogram (mg kg^-1^) FW.

Two grams of pummelo juice sac were homogenized with 8 mL of 1% HCl-methanol solution, followed by an extraction step at 4°C in the dark for 20 min, and vacuum filtered to remove the pumice. Following the Folin–Ciocalteu method and the AlCl_3_ colorimetric method outlined by Nxumalo et al. (2022), both TPC and total flavonoids content (TFC) were measured at 760 nm and 510 nm, with GA and rutin as the standard, respectively, and the results of both TPC and TFC were expressed as mg equivalent per 100 g (mg 100 g^-1^) of juice sac FW.

Two different assays were applied to assess the total antioxidant capacity in the juice sac of pummelo fruit: DPPH and hydroxyl radical (**·**OH) scavenging capacity assays. Determination of DPPH scavenging capacity was performed as described by Chen et al. (2022) with slight modifications. Briefly, 100 μL of the extracted juice sample was mixed with 1 mL of 0.1 mM DPPH solution, and allowed the mixture to stand in darkness for 30 min at 25°C before recording the absorbance at 517 nm. Adding 100 μL of deionized water in 1 mL of 0.1 mM DPPH solution was used as a control. The capacity to scavenge DPPH was expressed as percentage (%) and calculated by the following formula: (control OD_517_ - sample OD_517_)/control OD_517_ ×100.

The **·**OH scavenging capacity was determined based on the SA–Fenton method as described by Yun et al. (2021) with slight modifications. Briefly, the juice supernatant was extracted from 0.5 g of pummelo juice sac was sample with 5 mL of 50% (*v/v*) ethanol. The reaction system consisted of 0.5 mL of 9 mM iron sulfate, 0.5 mL of 9 mM SA (dissolved in ethanol), 0.5 mL of juice supernatant, 2.5 mL of deionized water and 0.5 mL of 8.8 mM H_2_O_2_. After the water bath at 37°C for 20 min, the absorbance of the mixture reaction was determine at 410 nm. The **·**OH scavenging capacity was expressed as percentage (%) and calculated with the formula as follows: (control OD_410_ - sample OD_410_)/control OD_410_ ×100.

### 2.7 Extraction and determination of ROS-scavenging enzymes activities

For the enzyme extraction and activity assay, all steps were carried out at 4°C. The extraction of crude enzyme was obtained by homogenizing 2.0 g of frozen juice sac powder with 8 mL of pre-cooled 100 mM phosphate buffer (pH 7.5, containing 5 mM DTT and 5% PVP) and removing the sediment by the centrifugation at 12 000 × *g* for 30 min, and the supernatant was collected for assaying ROS-scavenging enzymes [e.g., superoxide dismutase (SOD), catalase (CAT) and ascorbate peroxidase (APX), glutathione reductase (GR) and peroxide (POD)] activities.

SOD (EC 1.15.1.1) activity was determined *via* a specific SOD test kit (No: BC0170, Solarbio, Beijing, China) by detecting the absorbance of the reaction system at 560 nm. SOD activity was reported as U g^-1^, where one unit (U) of SOD activity was equal to the photochemical reduction of nitroblue tetrazolium inhibited by 50% per minute. The activities of CAT (EC 1.11.1.6), APX (EC 1.11.1.11), and GR (EC 1.8.1.7) were measured as the description of Ackah et al. (2022) and Peng et al. (2022), and the results were reported as U g^-1^ FW, where one unit (U) of CAT, APX, and GR activities was defined as an increase in the absorbance by 0.01 per minute at 240 nm, 290 nm, and 340 nm, respectively. POD (EC 1.11.1.7) activity was monitored in accordance with the guaiacol oxidation method as reported by Nxumalo et al. (2022) with some modifications. Then, 200 μL of 0.5 M H_2_O_2_ (diluted with 50 mM phosphate buffer) was adding to trigger the reaction mixture for POD activity containing 3 mL of 25 mM guaiacol solution and 0.3 mL of crude enzyme. POD activity was expressed as U g^-1^, where one unit (U) of POD activity was equal to the absorbance at 470 nm increased by 1 per minute.

### 2.8 RNA extraction and RT-qPCR analysis

The extraction of total high-quality RNA was carried out on 0.5 g of frozen juice sac powder from the control and 0.3% SA-treated pummelo fruit according to the cetyltrimethyl ammonium bromide (CTAB) method described by Landi et al. (2021). The integrity and quantification of the extracted RNA were determined using 1.0% agarose gel electrophoresis and based on the absorbance ratio at 260/280 nm in an ultramicro spectrophotometer (NanoDrop 2000, Wilmington, USA), respectively. The first-strand cDNA synthesis and qRT-PCR analysis of ROS-scavenging enzymes encoding genes were orderly performed following the procedures described by Chen et al. (2022). The *Actin* (Cg8g022300) gene was used as the internal control gene. Specific primers of *CmSOD* (Cg7g011780), *CmCAT* (Cg3g025260), *CmAPX* (Cg6g002810), *CmGR* (Cg5g018970), *CmPOD* (Cg2g001370), and *Actin* were designed with Primer 5.0 and listed in **Supplementary Table 1**. The transcript levels of the above-mentioned genes were quantified using the 2^-ΔΔCt^ method (Livak and Schmittgen, 2001).

### 2.9 Statistical analysis

**Figure 1:**
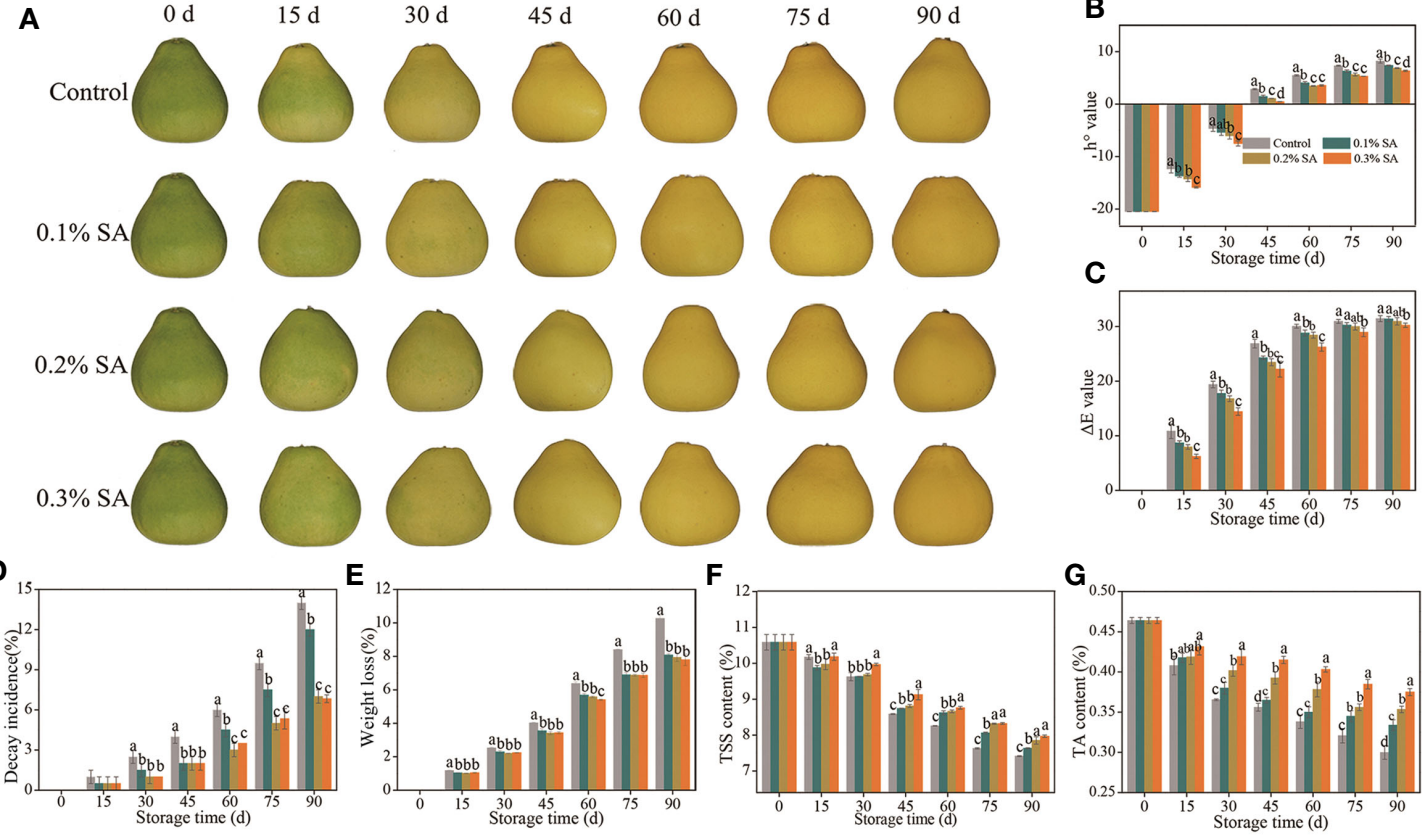
The mitigating effects of pre-storage SA treatment on color development **(A)**, decay incidence **(B)**, weight loss **(C)**, h° value **(D)**, ΔE value **(E)**, TSS content **(F)**, and TA content **(G)** of harvested ‘Jinshayou’ pummelo fruit. The different letters for each same sampling point indicate significant differences at *P*< 0.05 according to Duncan’s multiple range test.

**Figure 2:**
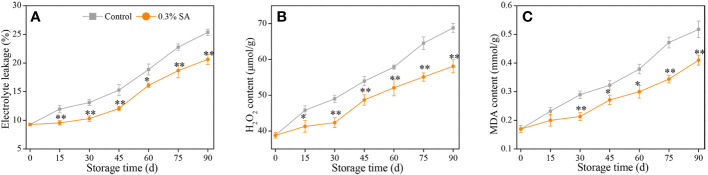
Variation in electrolyte leakage **(A)**, H_2_O_2_ content **(B)**, and MDA content **(C)** in the juice sac of ‘Jinshayou’ pummelo fruit treated with SA at 0.3% or untreated (control) during room temperature storage period. The asterisk of * (*P<* 0.05) or ** (*P<* 0.01) within the same sampling point denotes a significant difference between the control and 0.3% SA-treated group.

**Figure 3:**
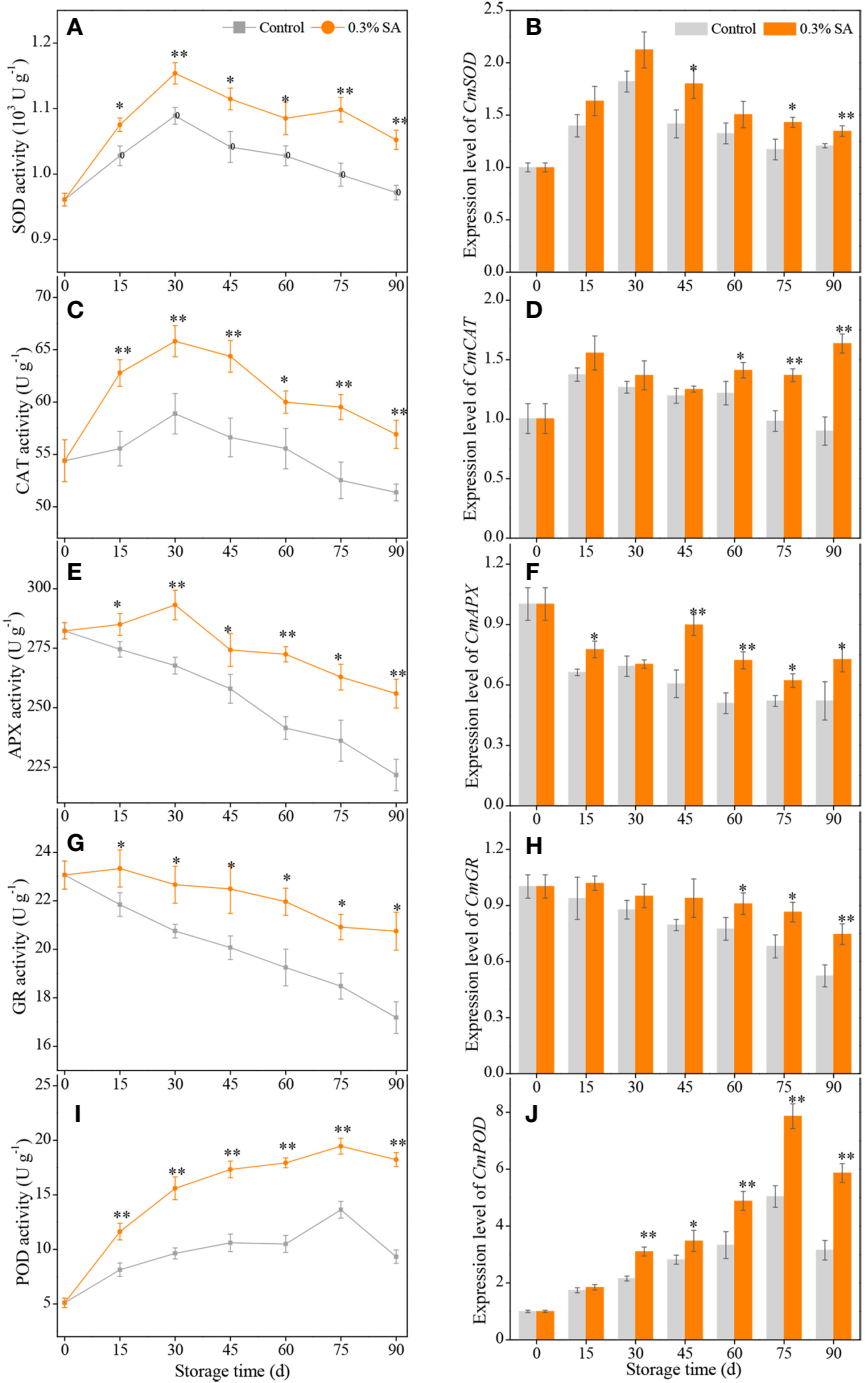
The activities and expression levels of SOD **(A, B)**, CAT **(C, D)**, APX **(E, F)**, GR **(G, H)**, POD **(I, J)** in the juice sac of ‘Jinshayou’ pummelo fruit treated with SA at 0.3% or untreated (control) during room temperature storage period. *β-actin* (Cg8g022300) was used as the internal control gene. The asterisk of * (*P<* 0.05) or ** (*P<* 0.01) within the same sampling point denotes a significant difference between the control and 0.3% SA-treated group.

**Figure 4:**
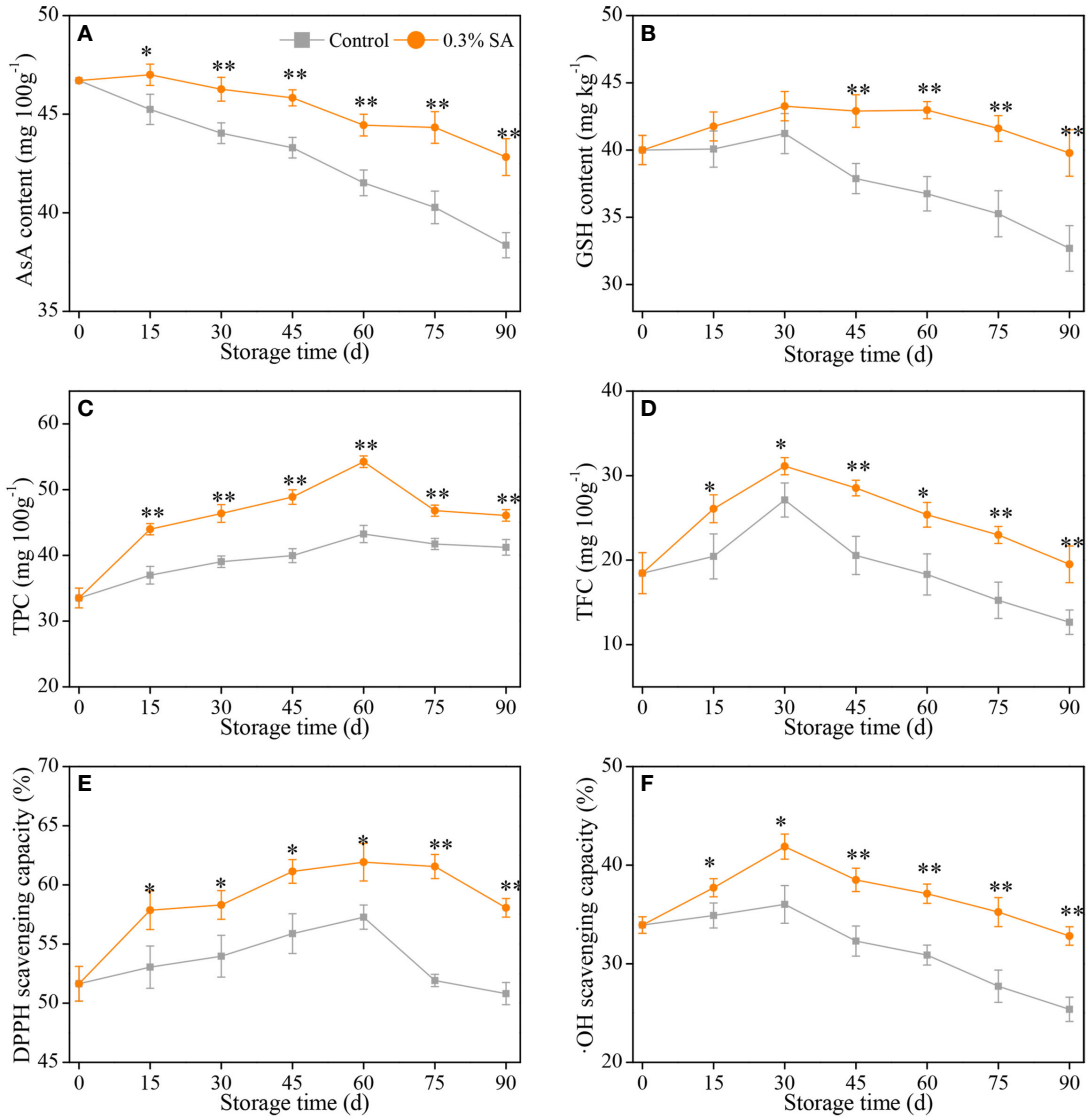
Variation in AsA content **(A)**, GSH content **(B)**, TP content **(C)**, TF content **(D)**, DPPH scavenging capacity **(E)**, and •OH scavenging capacity **(F)** in the juice sac of ‘Jinshayou’ pummelo fruit treated with SA at 0.3% or untreated (control) during room temperature storage period. The asterisk of * (*P<* 0.05) or ** (*P<* 0.01) within the same sampling point denotes a significant difference between the control and 0.3% SA-treated group.

**Figure 5:**
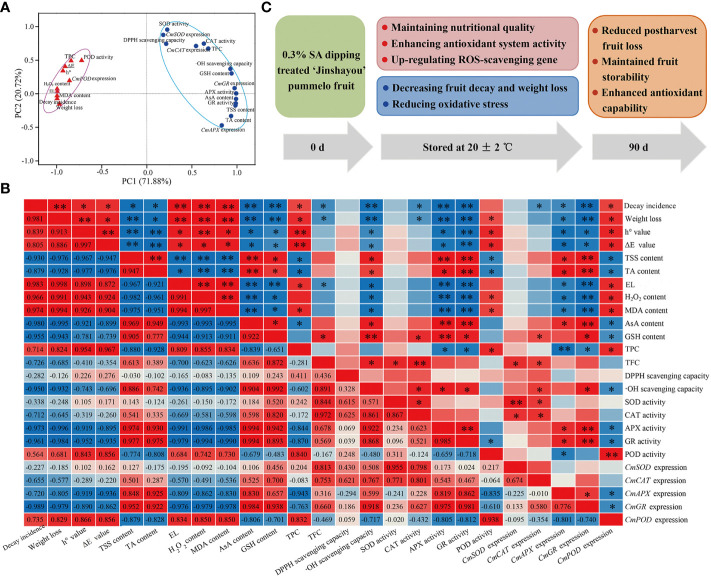
The PCA **(A)** and correlation analysis **(B)** of the ROS metabolism-related parameters in the control pummelo fruit stored at 20°C for 90 d. Correlation coefficients are shown in the lower triangle, while statistical significance (P < 0.05 or P < 0.01) is marked with * or ** in the upper triangle. A proposed model of the potential mechanism of pre-storage 0.3% SA treatment enhanced the storability and maintained higher redox state by regulating ROS-scavenging system in harvested ‘Jinshayou’ pummelo fruit **(C)**.

All physico-biochemical parameters and gene expression data were acquired from three biological replicates (n = 3), each comprising a separate mixture of 10 pummelo fruit sampled per replicate. Duncan’s multiple range test was applied to analyze the data using SPSS version 20.0. Significant differences among the control and SA treatments are highlighted with lowercase letters in **Figure 1**, and significant differences between both the control and 0.3% SA-treated fruit are highlighted with asterisks (*P< 0.05 or **P< 0.01) in **Figures 2**-**5**.

## 3 Results

### 3.1 Effects of postharvest SA treatment on color performance, decay rate, weight loss, TSS content and TA level

Color is a pivotal factor which influences the acceptability of the fruit by consumers. Surface color of ‘Jinshayou’ pummelo fruit turns from green to yellow during room temperature storage (**Figure 1A**). Both h° and ΔE values revealed a gradual increase in ‘Jinshayou’ pummelo fruit of three SA-treated and non-treated groups throughout the storage (**Figures 1B, C**). Nevertheless, the two increases in h° and ΔE value was significantly reduced by SA treatment in comparison with the control fruit surface. The inhibitory effect of pre-storage SA treatment was positively correlated with the SA-treated concentration, 0.3% SA-dipped treatment showed a significant delay on the increment of h° value in contrast with its 0.2% and 0.1% concentrations (**Figures 1A–C**).

A remarkable increase in decay incidence was observed in pummulo fruit with the prolongation of storage period from 30 to 90 d irrespective to the given SA-dipped treatment (**Figure 1D**). However, pre-storage treatment of SA dipping exhibited a significant inhibition in decay incidence compared to the control fruit throughout the storage period. This inhibitory effect until 90 d after the SA-dipped treatment, with the lowest decay incidence of 6.83% in the 0.3% SA-treated fruit, followed by the 0.2% and 0.1% SA-treated fruit, and finally the control (untreated) fruit, suggests that a beneficial effect of pre-storage treatment of SA dipping as a protective barrier avoiding postharvest pathogen infection or abiotic stress.

In general, fruit weight loss increased gradually throughout the storage period. The weight loss of pummulo fruit increased during the room temperature storage in case of all SA-dipped and control treatments (**Figure 1E**). Pre-storage treatment of SA dipping had no significant effect but delayed the weight loss percentage compared to the control fruit. At the end of storage (90 d), the highest weight loss of 10.27% was observed in control fruit, whereas the 0.3% SA-treated fruit showed the lowest weight loss of 7.61%.

The TSS content in pummelo fruit decreased continuously in all treatments, and this decline was effectively postponed by pre-storage treatment of SA dipping from 45 to 90 d (**Figure 1F**). At 90 d of storage, a reduction rate of TSS content reached 25.8% in the control fruit, while it was 22.5%, 20.0%, and 17.8% in 0.1%, 0.2%, and 0.3% SA-treated pummelo fruit, respectively. Significant differences were observed between the control and SA-treated fruit after 45 d of storage, with the most effective ability to reduce TSS degradation in the 0.3% SA-treated pummelo fruit. This characteristic of remained high TSS content is beneficial for maintaining the flavor quality and extending the storage life of pummelo fruit, because at the advanced stage of storage, once senescence stress has occurred, a rapid deterioration in fruit quality starts, resulting in serious quality losses.

At the beginning of storage (0 d), pummelo fruit showed the highest TA content (0.46%) which dropped continuously with the stretch of storage period. A rapid degradation of TA was recorded in the control fruit as compared to its slow degradation in 0.2% and 0.3% SA-treated fruit from 30 to 90 d (**Figure 1G**). The maximum TA loss in the control fruit was 34.8% compared to the minimum loss of 19.6% in pummelo fruit treated with 0.3% SA dipping. The decline of TA content in pummelo juice sacs was seen to be correlated to the senescence process of fruit, and the 0.3% SA-dipped treatment showed the most effective suppression of delaying the TA decrease.

### 3.2 Effects of postharvest SA treatment on oxidative stress

The parameters of EL and H_2_O_2_ content usually reflected the damage caused by oxidative stress in harvested fruits and vegetables (Nie et al., 2020; Ackah et al., 2022; Chen et al., 2022). Both EL and H_2_O_2_ content in harvested pummelo fruit showed a rising trend over the course of 90 d of storage (**Figures 2A, B**). However, the control pummelo fruit recorded higher increases of EL and H_2_O_2_ content than the 0.3% SA-treated fruit. Pummelo fruit treated with 0.3% SA exhibited 24.7% lower EL along with 15.9% lower H_2_O_2_ content, respectively, than the control one at the end of storage. In addition, MDA content is a target indicator used for the judgment of the membrane lipid peroxidation’s extent in plants under unfavorable conditions, including postharvest senescence stress (Ge et al., 2020; Sinha et al., 2022). As shown in **Figure 2C**, MDA content in the control fruit was displayed to gradually increase during the storage with its maximum level of 0.50 mmol g^-1^ at 90 d. MDA content in the 0.3% SA-treated fruit increased at a slower rate, with the overall MDA content being 19.7% lower than that of the control pummelo fruit from 30 to 90 d after the storage.

### 3.3 Effects of postharvest SA treatment on activities and expression levels of SOD, CAT, APX, GR and POD

During the storage at room temperature (20°C), ROS accumulation could lead to a decline in storage quality due to oxidative stress, as could the enzymatic antioxidant system, including SOD, CAT, APX, GR, and POD. As can be seen in **Figure 3A**, SOD activity in both treatments rose on the first 30 d of storage period and declined subsequently, but SOD activity in pummelo juice sac was enhanced by pre-storage 0.3% SA treatment, with 10.2% higher SOD activity than that in the control sample at 75 d. Pre-storage 0.3% SA treatment significantly increased the expression level of *CmSOD* at 45 d, 75 d, and 90 d, which were 26.8%, 22.2%, and 11.6% higher than those in the control pummelo juice sac, respectively (**Figure 3B**).

Two peaks of CAT activity in both treatments were assayed at 30 d of room temperature storage (**Figure 3C**). The maximum CAT activity in the 0.3% SA-treated pummelo juice sac was 1.13-fold higher compared with that in the control sample. Pre-storage 0.3% SA treatment also remarkably increased the expression level of *CmCAT* during the late 30 d of storage, which resulted in a higher overall *CmCAT* expression level by 42.7% compared to the control pummelo juice sac from 60 d to 90 d (**Figure 3D**).

APX activity in the control pummelo juice sac consistently decreased with the advancement of storage and reached its minimum value at the end of storage, while APX activity in the 0.3% SA-treated fruit slightly increased and peaked at 30 d, followed by a continuous decrease during the remaining storage period (**Figure 3E**). The levels of APX activity in the 0.3% SA-treated pummelo juice sac were much higher than those in the control over the entire storage period. Simultaneously, pre-storage 0.3% SA treatment remarkably up-regulated the expression level of *CmAPX* gene throughout the storage (except at 30 d time point, **Figure 3F**).

A gradual decrease in GR activity was observed in both treatments (**Figure 3G**). However, pummelo juice sac treated with 0.3% SA exhibited a higher GR activity during the entire storage period, resulting in a higher overall GR activity by 12.4% compared to the control treatment. The expression level of *CmGR* exhibited a trend similar to that of GR activity. Pre-storage 0.3% SA treatment prominently delayed the decline of *CmGR* expression level in pummelo juice sac during the late 30 d of storage period, with an overall *CmGR* expression level by 27.3% (**Figure 3H**).

POD activity in the control and 0.3% SA-treated pummelo juice sac increased gradually and reached the peak values of 13.62 ± 0.76 and 19.74 ± 0.92 U g^-1^ at 75 d and then dropped to 9.33 ± 0.61 and 18.23 ± 0.64 U g^-1^ at the end of storage (90 d), respectively (**Figure 3I**). Compared to the control treatment, significantly higher levels of POD activity were appeared in the 0.3% SA-treated pummelo throughout the room temperature storage. Additionally, a higher *CmPOD* expression level was displayed in the 0.3% SA-treated pummelo juice sac than the control, with a noticeable discrepancy in the middle and late storage (**Figure 3J**).

### 3.4 Effects of postharvest SA treatment on ROS-scavenging compounds and antioxidant capacity

AsA is considered as not only a key primary component affecting citrus quality, but also one of the endogenous non-enzymatic antioxidants involved in the clearance of ROS over-accumulation (Nie et al., 2020; Serna-Escolano et al., 2021; Ackah et al., 2022). As illustrated in **Figure 4A**, the variations of AsA content in pummelo juice sac from both groups showed a declining trend with the increase of storage duration, with a more pronounced decline in AsA content of the control pummelo juice sac. Furthermore, the 0.3% SA-treated pummelo fruit had a notably AsA content than the control throughout the whole of storage. In general, AsA content of room temperature-stored pummelo fruit was decreased with increased storage period, but its decline was considerably mitigated by 0.3% SA treatment. In this sense, 0.3% SA-treated pummelo fruit exhibited a less reduction in AsA content, which is probably because that pre-storage 0.3% SA treatment retarded the AsA oxidation in pummelo juice sac by the oxidative stress.

In addition to AsA, GSH is another representative substrate in AsA-GSH system (Halliwell-Asada cycle), both of them perform a pivotal role in the AsA-GSH system together with other enzymatic antioxidant systems to maintain redox homeostasis in postharvest fruits, and their amount can directly indicate the fruits’ ability to scavenge ROS (Hanaei et al., 2022; Peng et al., 2022). **Figure 4B** showed that GSH content in the control and 0.3% SA-treated fruit revealed the similar trend — i.e., a slight increase over the first 30 d of postharvest storage, followed by a gradual decrease. During the middle to late stage of storage, pre-storage 0.3% SA-dipped treatment remarkably delayed the decline of GSH content, which was 13.3% at 45 d, 16.9% at 60 d, 18.0% at 75 d, and 21.7% at 90 d higher compared with that in the control, respectively.

As the main secondary metabolites in plants, both phenolics and flavonoids are not only closely associated with the color conversion, flavor formation, and stress resistance of harvested fruits, but also protect them from over-produced ROS-caused oxidative damage (Ackah et al., 2022; Jiang et al., 2022). TPC is one of the important indexes to evaluate the antioxidant capacity of harvested fruits. Both groups reached a peak in TP content at 60 d, with that of the 0.3% SA-treated group being the highest level of 54.3 ± 0.9 mg/100g, while the highest level of TP content in the control group being 43.2 ± 1.3 mg/g, respectively (**Figure 4C**). As illustrated in **Figure 4D**, TFC of pummelo fruit remarkably rose on the first 30 d, and then decreased until to 90 d (the end of storage). It is worth to mention that pre-storage treatment of SA dipping remarkably delayed the decline of TF content, being an overall 1.36 times higher compared to the control pummelo fruit during the last 60 d of storage period. Similar to the overall variations of TPC and TFC, both DPPH and •OH scavenging capacity in two room temperature-stored pummelo juice sacs rose incrementally and peaked at 60 d and 30 d, respectively, followed by a decline (**Figures 4E, F**). Compared with the initial value at 0 d, the increases of two peak values in the 0.3% SA-treated pummelo juice sac was 9.0% and 17.3% higher than that in the control, showing that pre-storage 0.3% SA-dipped treatment had a positive influence on the increase of hydrophilic bioactive antioxidants and the maintenance of ROS-scavenging ability in ‘Jinshayou’ pummelo fruit during postharvest storage.

### 3.5 Correlation analysis

To understand the impact of 0.3% SA-dipped treatment in ‘Jinshayou’ pummelo fruit, both principal component analysis (PCA) and correlation analysis were applied to identify the ROS metabolism-related parameters that functionally referred to these measured parameters in pummelo fruit after 0.3% SA treatment. All 25 parameters linked to fruit postharvest ROS metabolism were mainly clustered into two main components (PC1: 66.719%; PC2: 20.72%, **Figure 5A**). The PC1 consisted of 10 parameters, including fruit decay rate, weight loss, h° value, ΔE value, electrolyte leakage, H_2_O_2_ content, MDA content, TPC, POD activity, and *CmPOD* expression (**Figure 5A**). PC2 showed a higher relationship factor for five nutritional/functional components (TSS, TA, AsA, GSH, and flavonoids) contents, DPPH scavenging capacity, •OH scavenging capacity, four ROS-scavenging enzymes activities, and their encode gene expression levels, showing a high negative correlation to postharvest storability loss (**Figure 5B**; *P*< 0.05 or 0.01). The loss of fruit postharvest storability is due to the imbalance of ROS homeostasis, and result in an increase of defective fruit as well as the degradation of fruit quality. Pre-storage SA treatment was effective to enhance the antioxidant capability and reduce postharvest loss of pummelo fruit during room temperature storage. A similar finding was reported by Nie et al. (2020) in that chitosan-coated treatment could maintain high levels of some antioxidants (enzymes: APX, GR, SOD, and CAT) and reduce the oxidative stress. PCA can be an effective means to assess the preservative effect of pre-storage SA treatment on ‘Jinshayou’ pummelo fruit as demonstrated by other reports in other horticultural products (Bakpa et al., 2022; Nxumalo et al., 2022). In the present study, fruit decay incidence was highly positively correlated with electrolyte leakage, H_2_O_2_ content, and MDA content, with the coefficients being 0.983, 0.966, and 0.974, respectively (**Figure 5B**, *P<* 0.01), because it is highly related to oxidative stress and ROS accumulation. Moreover, ROS-scavenging enzymes activities (SOD, CAT, APX, and GR) and expression levels of their encoding genes (*CmSOD*, *CmCAT*, *CmAPX*, and *CmGR*) exhibited high negative correlations with fruit decay rate and oxidative stress-related parameters (*P*< 0.05 or 0.01). Other studies have also proved that ROS accumulation leads to membrane lipid deterioration and is highly negatively correlated with the activities of antioxidant enzymes during longan fruit storage (Chen et al., 2020). These findings well demonstrated that pummelo postharvest senescence was accompanied by a disability in antioxidant system and reduction in the overall quality, thus corroborating the intimate association between ROS metabolism and postharvest storability in ‘Jinshayou’ pummelo fruit during room temperature storage.

## 4 Discussion

Pummelo fruit, being a typical non-climacteric variety, is prone to a rapid decline in nutritional quality when stored at room temperature, thus limiting its storability. Numerous studies have revealed that pre-storage SA treatment prior to storage can delay postharvest senescence and quality deterioration of a variety of horticultural fruits, including apricot, blueberry, cornelian cherry, goji berry, papaya, pear, winter jujube, and tomato, as well as improve their resistance to abiotic stress (Dokhanieh et al., 2013; Hanif et al., 2020; Kumar et al., 2021; Zhang et al., 2021a; Jiang et al., 2022; Li et al., 2022; Sinha et al., 2022; Zhang et al., 2022). In this study, pre-storage application of SA was found to effectively in delaying fruit color change and decreasing h° and ΔE values, as well as reducing fruit decay and weight loss of harvested ‘Jinshayou’ pummelo fruit during storage at room temperature (**Figures 1A–E**). Pre-storage SA treatment at 0.3% was the more effective in terms of prolonging the storage duration to 90 d at 20°C. The protection of harvested fruit from color change and the inhibition of postharvest loss were observed when exogenous SA application was applied in varying concentrations; such as 2.0 mM SA on ‘Patharnakh’ pear (Adhikary et al., 2021), 0.5 mM SA on ‘Taaptimjaan’ wax apple (Supapvanich et al., 2017), and 0.05% SA on ‘Dongzao’ winter jujube (Zhang et al., 2022).

The deterioration of horticultural fruits’ nutritional quality, mainly due to their own respiration rate, is a major cause for fruit flavor loss and is regarded as a key metric to evaluate their storability and postharvest freshness during storage (Lacerna et al., 2018; Haider et al., 2020; Nie et al., 2020). As harvested fruits undergo senescence, the soluble sugar and organic acids gradually degrade, thus delaying this process can help to maintain the fruit’s flavor quality and extend its storage life (Chen et al., 2005; Baswal et al., 2020; Serna-Escolano et al., 2021). Both TSS and TA contents are essential metrics for assessing the maturity and ripeness process of horticultural fruit, particularly citrus fruit, which mainly determine the storability and overall flavor (Huang et al., 2021; Nxumalo et al., 2022). The data depicted in **Figures 1F, G** revealed that pre-storage 0.3% SA treatment can reduce the drop in TSS and TA content of ‘Jinshayou’ pummelo fruit throughout the storage period, as both levels are higher than the control and other two SA-treated fruit, which were in accordance with pre-storage treatment of SA to ‘Kinnow’ mandarin (Haider et al., 2020), ‘Patharnakh’ pear (Adhikary et al., 2021), ‘Sabrosa’ strawberry (Asghari and Hasanlooe, 2015), and ‘Tupi’ blackberry (Martínez-Camacho et al., 2022). Moreover, it was noted that 0.3% SA treatment displayed the most effective delay in the degradation of TSS and TA, from which the highest levels of TSS and TA content was obtained over the entire storage period.

Citrus fruits are stored at room temperature for too long, resulting in a loss of cell membrane integrity, which can be determined through EL, MDA accumulation and H_2_O_2_ production (Hanaei et al., 2022; Martínez-Camacho et al., 2022; Nxumalo et al., 2022). Prolonged exposure to senescence-elicited oxidative stress results in membrane lipid peroxidation. It is universally acknowledged that ROS-induced oxidative stress is a crucial factor leading to storability loss and quality deterioration of harvested fruits. ROS, especially H_2_O_2_, can cause the oxidation of unsaturated fatty acids, disrupting the cell membrane’s integrity, eventually leading to membrane lipid peroxidation (Chen et al., 2020; Sinha et al., 2022). In pummelo fruit, the levels of electrolyte leakage, H_2_O_2_ content and MDA content in both treatments increased dramatically from 30 d onwards (**Figure 2**). Fruit senescence process advances due to oxidative stress caused by excessive ROS accumulation, which concomitantly give out a large amount of free radicals as by-products. In ‘Majiayou’ pummelo with red flesh, the accumulations of superoxide anions, H_2_O_2_, and MDA during room temperature storage was also reported by Nie et al. (2020), who pointed out that postharvest 1.5% chitosan treatment could reduce juice sac granulation stress by delaying ROS accumulation. Zhang et al. (2022) also ascertained that pre-storage treatment with 0.05% SA had a direct or indirect effect on the retardation of fruit respiration rate and senescence process, thereby decreasing the accumulation of ROS and maintaining postharvest quality of winter jujube. Pre-storage SA treatment also demonstrated its extraordinary capacity to scavenge over-produced ROS by enhancing the antioxidant system under oxidative stress.

It is a well-established notion that the maintenance of ROS homeostasis could decrease excess ROS accumulation and protects living cells from oxidative stress damage, which is vital for enhancing postharvest storability and maintaining overall quality in harvested fruits (Asghari and Hasanlooe, 2015; Huang et al., 2021; Ackah et al., 2022). SOD, CAT, APX, GR, and POD are pivotal antioxidant enzymes that share their responsibility for scavenging excess ROS, thus protecting plant cells against oxidative stress (Marschall and Tudzynski, 2016; Nie et al., 2020; Chen et al., 2022). Specially, SOD plays its pioneer role in the ROS-scavenging enzymatic antioxidant system, which can dismutate superoxide anion into O_2_ and H_2_O_2_; subsequently, the disproportionated H_2_O_2_ is directly or indirectly decomposed into O_2_ and H_2_O_2_ with the concerted effort from CAT, APX, GR, and POD (Siboza et al., 2017; Martínez-Camacho et al., 2022; Peng et al., 2022). Numerous studies have proved that pre-storage SA treatment helps in maintaining or enhancing the antioxidant capacity in harvested fruits through the activation of antioxidant enzymatic system (Dokhanieh et al., 2013; Serna-Escolano et al., 2021; Zhang et al., 2021a). To reduce ROS-induced oxidative damage, plant cells initiate an enzymatic antioxidant system as a self-protective response. In this study, the higher levels of SOD, CAT, APX, GR, and POD activities was observed in the 0.3% SA-treated pummelo fruit; furthermore, the expression levels of these genes encoding *CmCAT*, *CmAPX*, *CmGR*, and *CmPOD* were up-regulated by 0.3% SA treatment in pummelo fruit (**Figure 3**), accompanying by the inferior H_2_O_2_ content and the lower MDA accumulation (**Figure 2**). The elimination of H_2_O_2_ and the less level of oxidative stress in pummelo juice sacs are dependent on the improvement of SOD, CAT, POD, APX, and GR activities, and the up-regulation of their encoding gene expressions. Other reports also indicated that elevated ROS-scavenging enzyme activities and up-regulated their encoding gene expressions contributed to the balance of ROS homeostasis and the maintenance of postharvest storability in ‘Eureka’ lemon (Siboza et al., 2017), ‘Dahuang’ apricot (Wang et al., 2015), ‘Ningqi No. 5’ goji berry (Zhang et al., 2021a), ‘Cornelian’ cherry (Dokhanieh et al., 2013), and ‘Dongzao’ winter jujube (Sang et al., 2022), and ‘Majiayou’ pummelo (Nie et al., 2020). Above findings explicitly showed that the maintained storability by exogenous SA treatment might be correlated with improvement of the fruit’s own antioxidant enzymatic system. The findings in the present study plainly indicated that pre-storage 0.3% SA-dipped treatment increased ROS-scavenging enzymes activities and simultaneously up-regulated their encoding genes expression, thereby reducing ROS-caused oxidative stress in pummelo juice sacs. Thus, it could be inferred that 0.3% SA treatment contributed to the active function of enzymatic antioxidant system in ‘Jinshayou’ pummelo fruit, minimized the ROS-induced oxidative stress, and enhanced or maintained stress resistance in pummelo juice sacs, thereby reducing postharvest loss and extending storage life.

In addition to enzymatic antioxidant system, the non-enzymatic antioxidant system also has its irreplaceable role in reducing the oxidative senescence in harvested fruits (Supapvanich et al., 2017; Hanif et al., 2020; Nie et al., 2020). In higher plants, AsA and GSH are both representative substrates in AsA-GSH system (Halliwell-Asada cycle) for scavenging over-accumulated ROS in fruit, which maintains ROS homeostasis and delays cell senescence caused by oxidative stress (Serna-Escolano et al., 2021; Hanaei et al., 2022; Peng et al., 2022). Additionally, phenolics compounds are a class of plant secondary metabolites that perform a pivotal role in the color conversion, flavor formation, and stress resistance of harvested fruits, in conjunction with flavonoids, which protect them from the oxidative damage caused by the over-production of ROS (Ackah et al., 2022; Jiang et al., 2022). Therefore, high levels of antioxidants (AsA, GSH, phenolics, and flavonoids) contents are closely related to the fruit’s resistance to postharvest senescence stress. This study showed that pummelo juice sacs treated by 0.3% SA dipping presented the higher levels of AsA content, GSH content, TPC, and TFC (**Figures 4A–D**) accompanied by superior DPPH and •OH radicals scavenging capacity (**Figures 4E, F**), which may occur thanks to higher levels of ROS-scavenging enzymes activities along with their encoding gene expression during room temperature storage (**Figure 3**). Haider et al. (2020) and Zhang et al. (2021b) found that high levels of AsA, phenolics and flavonoids were beneficial to enhance the antioxidation capacity of ‘Kinnow’ mandarin and ‘Ningqi No. 5’ goji berry to postharvest oxidative stress. Similar findings were also obtained for pre-storage SA treatment in ‘Fino’ lemon (Serna-Escolano et al., 2021), ‘Cornelian’ cherry (Dokhanieh et al., 2013), and ‘Sabrosa’ strawberry (Asghari and Hasanlooe, 2015). Therefore, our results showed that pre-storage 0.3% SA-dipped treatment had a positive influence on the increase of hydrophilic bioactive antioxidants, the improvement of ROS-scavenging ability to resist oxidative stress, the maintenance of overall quality, and the extension of the shelf life of ‘Jinshayou’ pummelo fruit.

## 5 Conclusion

In summary, pre-storage treatment of 0.3% SA dipping was found be to effective in reducing postharvest fruit loss and maintaining the overall quality of ‘Jinshayou’ pummelo fruit. This was mainly due to 0.3% SA enhancing the activities of ROS-scavenging enzymes, along with up-regulating their corresponding gene expressions, maintaining higher levels of TSS, TA, AsA, GSH, TFC, and free radical scavenging capacity, and decreasing fruit decay incidence. All of these contributed to reducing oxidative stress and stabilizing the ROS homeostasis in pummelo juice sacs, thereby maintaining postharvest storability and overall quality in ‘Jinshayou’ pummelo fruit (**Figure 5C**). These results indicate that pre-storage 0.3% SA treatment could potentially provide a feasible preservation technology in reducing postharvest loss and maintaining ROS metabolism, and thus delay postharvest senescence and extend storage life of ‘Jinshayou’ pummelo fruit after harvest.

## Data availability statement

The original contributions presented in the study are included in the article/**Supplementary Material**. Further inquiries can be directed to the corresponding author.

## Author contributions

QH: methodology, formal analysis, data curation, and writing-original draft. LH: methodology, investigation, and validation. JC: conceptualization, supervision, and funding acquisition. YZ: methodology, software, and investigation. WK: investigation and formal analysis. CC: conceptualization, supervision, project administration, and writing-review and editing. All authors contributed to the article and approved the submitted version.
